# Exosomal lncRNA 91H is associated with poor development in colorectal cancer by modifying HNRNPK expression

**DOI:** 10.1186/s12935-018-0506-2

**Published:** 2018-01-23

**Authors:** Tianyi Gao, Xiangxiang Liu, Bangshun He, Zhenlin Nie, Chengbin Zhu, Pei Zhang, Shukui Wang

**Affiliations:** 1 Department of Clinical Laboratory, Nanjing First Hospital, Nanjing Medical University, 68 Changle Road, Nanjing, 210006 Jiangsu China; 2 Central Laboratory, Nanjing First Hospital, Nanjing Medical University, 68 Changle Road, Nanjing, 210006 Jiangsu China

**Keywords:** Exosomes, lncRNA 91H, HNRNPK, Colorectal cancer (CRC), Prognosis

## Abstract

**Background:**

Exosomes mediated transfer of lncRNA 91H may play a critical role in the development of CRC. However, few studies have proved the mechanism. So we performed this study to deeply explore the biological functions of exosomal 91H in the development and progression of CRC.

**Methods:**

The association between lncRNA 91H and exosomes was detected in vitro and vivo. Then RNA pulldown and RIP were used to detect how lncRNA 91H affect CRC IGF2 express. At last, clinic pathological significance of exosomal 91H was evaluated by Cox proportional hazards model.

**Results:**

We found that serum lncRNA 91H expression was closely related to cancer exosomes in vitro and vivo which may enhance tumor-cell migration and invasion in tumor development by modifying HNRNPK expression. Then the clinic pathological significance of exosomal 91H was evaluated which demonstrated that CRC patients with high lncRNA 91H expression usually showed a higher risk in tumor recurrence and metastasis than patients with low lncRNA 91H expression (P < 0.05).

**Conclusion:**

All these data suggested that exosomal lncRNA 91H enhancing CRC metastasis by modifying HNRNPK expression might be an early plasma-based biomarker for CRC recurrence or metastasis. Further large-scale studies are needed to confirm our findings.

## Background

Colorectal cancer (CRC) is one of the leading causes of cancer-related morbidity and mortality [1]. Numerous studies suggested that the first stage of metastasis occurs early and more than 60% of patients have initiated the metastatic process by the time of diagnosis [2]. Many genetic factors has been identified to be involved in CRC development and the identification of such factors may improve the prevention of this malignancy and reduce mortality [3].

Previous studies have indicated that long noncoding RNAs (lncRNAs) is defined as transcripts with a minimum length of 200 nucleotides in size and limited protein-coding potential, which deregulated expression are implicated in the tumor formation and progression [4–6]. lncRNA 91H, a novel 119.392-kb long noncoding antisense transcripts, is located at the position of the H19/IGF2 locus (Accession Number NC_000011.9). It has been proved to be consistently overexpressed in a number of human cancer tissues, including CRC which is significantly associated with adverse clinical characteristics and plays an important role in tumor development [7–9]. However, its specific mechanism and the association with tumor progression still remains unclear.

Exosomes are 30–150 nm sized membranous vesicles that are endogenously produced by almost all cell types which could participate in intercellular communication by delivering proteins, miRNA, mRNA, and lncRNA to recipient cells [10, 11]. Emerging evidence has shown that exosomes play critical roles in causing deregulated local and systemic cellular communication in the tumor microenvironment. Recent studies have also demonstrated that by being assembled into cancer exosomes, lncRNAs could be secreted from tumor cells into body fluids such as blood, urine, milk, and saliva [12, 13]. These tumor-derived exosomes in turn enhanced tumor cell invasiveness and metastasis [14]. Pan et al. showed that in human gastric cancer (GC), exosomes mediated transfer of lncRNA ZFAS1 can enhance GC cell proliferation and migration [15]. In addition, it was also proved that lncRNA HOTAIR from patients with high-grade muscle-invasive disease are enriched in exosomes than other patients in urothelial bladder cancer [16].

In light of these observations, exosomal 91H might also play a critical role in the development of CRC. However, few studies have investigated the potential association in CRC. So we performed this study to deeply explore the biological functions of exosomal 91H in CRC development and progression.

## Materials and methods

### Patients

Serum samples from 232 CRC patients and 50 healthy people who underwent a routine health checkup and showed no disease at Nanjing First Hospital Affiliated to Nanjing Medical University, between 2011 and 2014 were collected in this study. Among serum samples, 68 were patients who were diagnosed as CRC without treatment, 56 were patients who had just underwent surgery without chemotherapy or radiotherapy and 108 were other patients who had underwent operation for more than half a year. Clinical information of all patients were collected including age, sex, TNM stage, tumor grade and family history of disease.

### Cell line authentication

Cell lines HCT-8, HCT-116 and FHC (normal human intestinal epithelial cell line) were directly obtained from Shanghai Cell Collection, Chinese Academy of Sciences on March, 2017. All above cell lines were maintained in DMEM (Hyclone, USA) with 10% fetal FBS (Gibco, MA, USA) and cultured at 37 °C with 5% CO_2_. All the tumor cell lines had been tested by short tandem repeat (STR) method in ABI 3500 Genetic Analyzer (USA Life 3500).

### Isolation of exosome

Serum and cell medium were treated with polyvinylidene fluoride filter (Millipore, Billerica, Mass). Then subsequently ExoQuick solution (System Biosciences, Mountain View, CA) was added incubating at room temperature for 0.5 h. By centrifugation at 1500*g* for 30 min. Exosome pellets were collected and resuspended in 25 μl DEPC water.

### Transmission electron microscopy

0.5 mg/ml exosomes spotted onto a glow-discharged copper grid on the filter paper were dried for 10 min by the infrared lamp. Then exosomes were stained in a drop of 1% aqueous solution of phosphotungstic acid for 6 min and dried for 25 min by the infrared lamp. Finally, exosomes were examined under transmission electron microscopy at 100 keV.

### Western blot analysis

Exosomes samples were lysed using a lysis buffer. Then, the protein concentration was quantified using a BCA Protein Assay Kit (Beyotime, Shanghai, China). Sodium dodecyl sulfate–polyacrylamide gel electrophoresis and western blot analyses were performed according to the standard procedures. Primary antibodies used included anti-HNRNPK (G. Dreyfuss, UPENN, Philadelphia, PA), anti-CD63 (sc5275, Santa Cruz Biotechnology, Santa Cruz, CA, USA) and anti-β-actin (1: 2000, ab8227, Abcam, Cambridge, UK). Secondary antibodies were goat anti-rabbit HRP secondary antibody (System Biosciences, Mountain View, CA).

### RNA pulldown and mass spectrometry analysis

LncRNA 91H RNAs in vitro were transcribed using the Biotin RNA Labeling Mix (Roche) and SP6 RNA polymerase (Ambion) and purified by RNA Clean & Concentrator™-5 (Zymo Research). Then cell lysates were freshly prepared using Protea Prep Zwitterionic Cell Lysis Kit, Mass Spec Grade (Protea^®^) with Anti-RNase, Protease/Phosphatase Inhibitor Cocktail, Panobinostat and Methylstat supplemented in the lysisbuffer. The BcMag™ Monomer avidin Magnetic Beads (Bioclone) were firstly prepared according to manufacturer’s instructions and then immediately subjected to RNA (20 µg) capture in RNA capture buffer for 25 min at room temperature with agitation. The RNA-captured beads were washed with NT2 buffer and incubated with 30 mg cell lysates diluted in NT2 buffer supplemented with 50 U/ml RNase OUT™, 50 U/ml Superase1·IN™, 2 mM dithiothreitol, 30 mM EDTA and Heparin 0.02 mg/ml at 4 °C for 2 h with rotation. Washing with NT2 buffer, NT2-high salt buffer (500 mM NaCl), NT2-high salt buffer (1 M NaCl), NT2-KSCN buffer (750 mM KSCN) for twice and PBS (once) for 5 min at 4 °C and eluted by 2 mM D-biotin in PBS. The eluted protein complexes were denatured, reduced, alkylated and digested with immobilized trypsin (Promega) for MS analysis at MD Anderson Cancer Center Proteomics Facility.

### Extraction of nucleic acids

RNA of cell lines, serum and exosome samples were extracted with E.Z.N.A. Total RNA Kit (omega, USA) following the manufacturers’ protocol. To avoid the possible DNA contamination, RNA was digested with RNase free DNaseI and cleaned with the RNA easy MinElute Cleanup Kit (Qiagen).

### RNA interference

RNA interference was carried out by using synthetic siRNAs, as described by earlier studies [17]. Two synthetic siRNAss (si-lncRNA 91H1 and si-lncRNA 91H2) corresponding to the lncRNA 91H RNA sequences 5′-GGCGUCAUUCUGAUGGGACTT-3′ and 5′-UUCAGGAGCUUAAGAUGCUTT-3′ were used and 5′-CGUGGGUGGAUGCAUGGAUTT-3′ was used as a negative control. The cells (5 × 10^2^) were grown on coverslips in six-well plates and transfected with 400 pmol of different siRNAs. After transfection, followed by lysed for total RNA isolation.

### Reverse transcription and real-time RT-PCR

Total reverse transcriptions were performed as follows: 1ug RNA, 4 µl of buffer containing random hexamers and reverse transcriptase (RT; Takara Japan) were incubated for 15 min at 37 °C, for 5 s at 85 °C, and stored at 4 °C in a final volume of 20 µl. Real-time PCR amplifications were performed by using a QuantiTect Sybrgreen PCR Kit (Qiagen) with 2 µl of cDNA and 500 nM concentrations of the primers. The primers used for the lncRNA 91H were Fw 5′-CCTGAAACAGGGAGGTGGTG-3′ and Rev 5′-GTGCCTGGAGCCTGTCTAAC-3′. We selected β-actin, a housekeeping gene as an internal control: Fw: 5′-CTCCATCCTGGCCTCGCTGT-3′ and Rev: 5′-GCTGTCACCTTCACCGTTCC-3′. Relative quantification of gene expression changes was recorded after normalizing for 18S expression, computed by using the 2^−∆∆CT^ method (user manual 2, ABI 7500 System).

### Scratch wound assay

Infected HCT-116 cells were cultured in six-well plates for 48 h. Then wounds were created in confluent cells using a 20 μl pipette tip. Free-floating cells and debris were removed using PBS. Then the cells were divided into 3 groups: cancer exosomes, cancer exosomes with lncRNA 91H interference and negative control without exosomes and FBS-free medium was added with or without exosomes (2 μg/µl). Finally we observed the wound healing at 0, 48 h and photographed representative scrape lines using the light microscope. Each experiment was repeated in triplicate.

### Migration and invasion assays

For migration assay, HCT-116 cells were divided into 3 groups: cancer exosomes, cancer exosomes with lncRNA 91H interference and negative control without exosomes. Then cells (1 × 10^5^) were seeded into the upper chamber of transwell plates with serum-free DMEM and exosomes (2 μg/µl), in a 24-well format with 6 mm diameters. 600 μl DMEM with 5% FBS were added to the bottom chamber. Culturing for 24 h, cells were fixed with methanol and stained with Wright’s stain. The top of the permeable membrane cells remained were removed using a cotton swab. At last the numbers of migrating cells were counted in five random fields under a light microscope and the average value of five fields was used.

For invasion assay, the top chambers were treated with 40 mg/ml basement membrane Matrigel (Becton–Dickinson, USA) at 37 °C for 30 min. As before, Cells treated with or without exosomes (1 × 10^5^) were added into the top chambers, the bottom chambers were filled with DMEM containing 10% FBS. After incubating for 24 h, the cells that invaded the reverse side of top chambers were fixed, stained, and counted using a light microscope. Each invasion assay was performed in three replicates.

### Statistical analysis

The cut-off levels of exosomal 91H the receiver operating characteristic curve analysis (ROC). Comparison of continuous data was analyzed using an independent t test, and categorical data were analyzed by Chi square test. The impact of exosomal 91H expression and clinicopathologic parameters on relapse-free survival (RFS) was calculated by using a Cox proportional hazards model. Statistics with P value < 0.05 were considered as statistically significant. All these statistical calculations were performed using IBM SPSS 22.0 software (IBM, USA).

### Equipment and settings

**Fig. 1:**
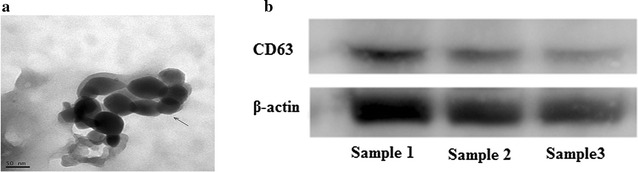
The transmission electron microscopy and western blot analysis of exosomes performed in the serum of CRC patients. **a** the picture of transmission electron microscopy of exosomes in CRC patients’ serum. **b** CD63, as a exosome marker, highly expressed in CRC patients’ serum

Exosomes in Fig. 1 were created by Gatan digitalmicrograph and Gels/blots were created by Syngene G:BOX. Photos of scratch wound and migration/invasion assays were all crested by Leica Application Suite. At last, we confirmed that all methods were performed in accordance with the relevant guidelines and regulations as described above.

## Results

### Characterization of exosomes in serum

The characteristics of exosomes in the serum were observed under transmission electron microscope (TEM) which exhibited double layer membrane that ranged from 40 to 100 nm (Fig. 1a). In addition, CD63 which was the protein marker of exosome, was also used to confirm the evaluation of exosomes by western blot analysis (Fig. 1b).

### The association between lncRNA 91H and exosomes

**Fig. 2:**
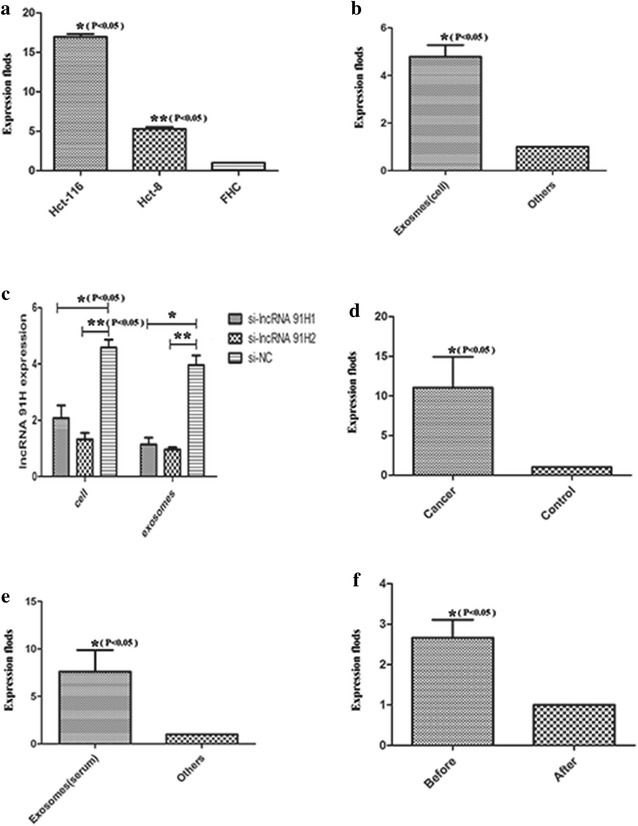
Exosomal 91H expression in CRC cells and cancer patients’ serum compared with negative control. **a** Exosomal 91H expression in CRC cell lines and a normal human intestinal epithelial cell line by qRT-PCR (P < 0.05). **b** lncRNA 91H expression in exosomes and other places of cell medium without exosomes by qRT-PCR (P < 0.05). **c** HCT-116 cells were transfected with siRNA-91H1, siRNA 91H2 and siRNA-NC. After 48 h, exosomal 91H expression was effectively inhibited by qRT-PCR compared with siRNA-NC (P < 0.05). **d** Exosomal 91H expression in CRC patients and healthy people by qRT-PCR (P < 0.05). **e** lncRNA 91H expression in exosomes and other places of CRC patients’ serum without exosomes by qRT-PCR (P < 0.05). **f** Exosomal 91H expression in CRC patients before and after operation

By performing qRT-PCR, the expression of exosomal 91H in cell lines HCT-8, HCT-116 were 16.96 and 5.30-fold change higher than FHC with P < 0.05 (Fig. 2a). Then the expression of lncRNA 91H in exosomes was compared with other places of HCT-116 medium which demonstrated that the expression of lncRNA 91H in exosomes was 4.72-fold change higher than other places of cell medium (Fig. 2b, P < 0.05). At last, RNA interference was used in this study. By transfection for 48 h, lncRNA 91H was effectively silenced in HCT-116 cells and the exosomal 91H was also decreased in each siRNA group compared with negative control (Fig. 2c, P < 0.05).

The association between lncRNA 91H and exosomes were also detected in 68 CRC patients without treatment. The RT-PCR products of lncRNA 91H extracted from part of cancer patients’ exosomes were firstly electrophoresed on 2% agarose gel with the length of 271 kb. Then by qRT-PCR, we found that the expression of exosomal 91H in patients’ serum was significantly higher than healthy people (Fig. 2d, P < 0.05). Next the lncRNA 91H expression in exosomes and serum without exosomes was compared with the result that the expression levels of lncRNA 91H in exosomes was significantly higher than other places of patients’ serum (Fig. 2e, P < 0.05). At last, the exosomal 91H expression in patients with operation were detected which showed that exosomal 91H expression was apparently decreased after operation (Fig. 2f, P < 0.05).

### Effect of lncRNA 91 on HNRNPK expression

**Fig. 3:**
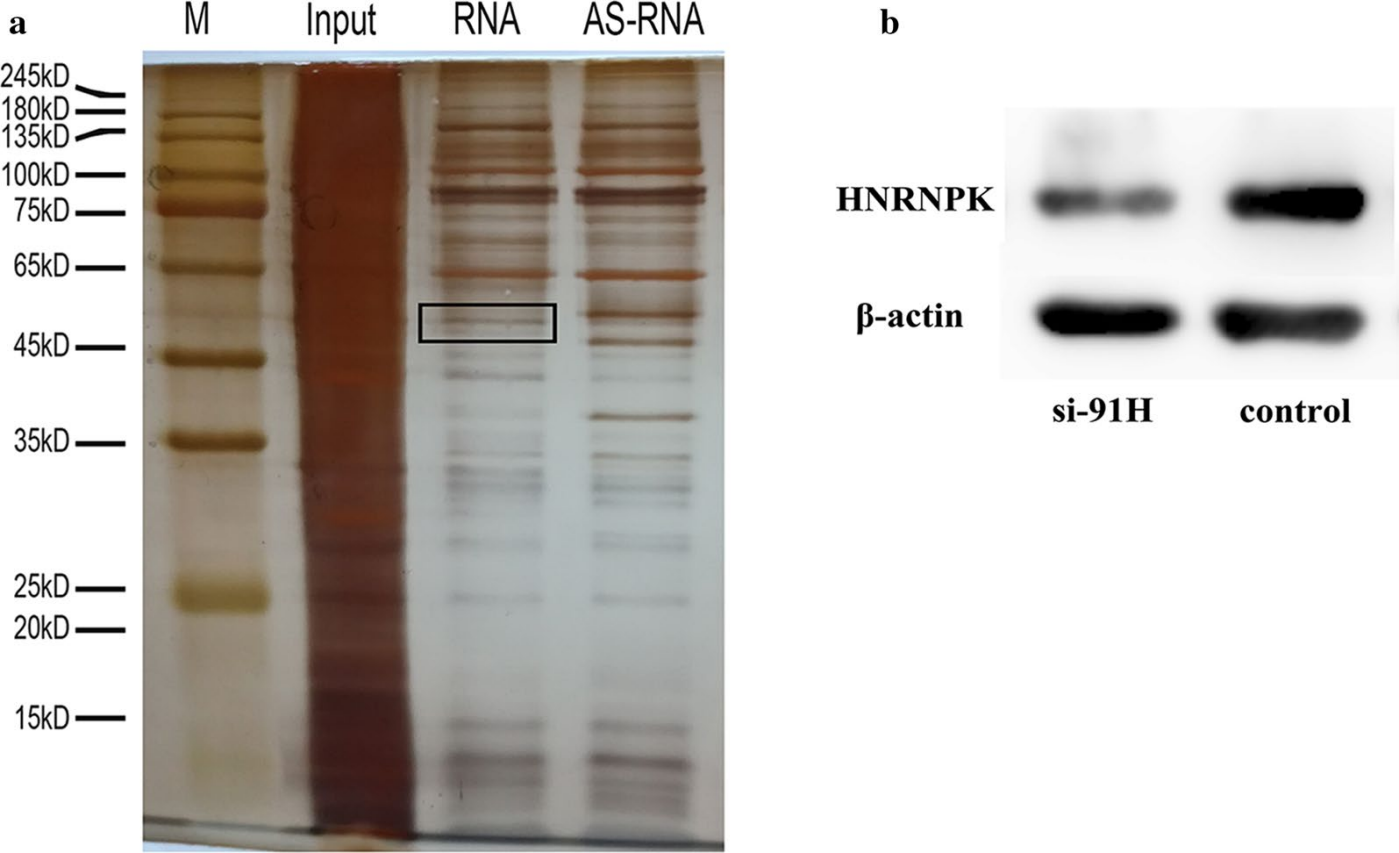
Effect of lncRNA 91 on HNRNPK expression. **a** Pull down results of lncRNA 91H by Silver staining. **b** HNRNPK expression levels of CRC cells treated with lncRNA 91H siRNA and negative control

We next investigated the molecular mechanism by which lncRNA 91 regulated tumor development. Pull down assays with cell lysate showed that heterogeneous nuclear ribonucleoprotein K (HNRNPK) protein might directly interact with lncRNA 91 by MS analysis (Fig. 3a). To further confirm the lncRNA 91H-protein interactions in vivo, RNA interference was used in this study which demonstrated that the HNRNPK protein expression significantly decreased compared with negative control (P < 0.05, Fig. 3b).

### Biological functions of exosomal 91H

**Fig. 4:**
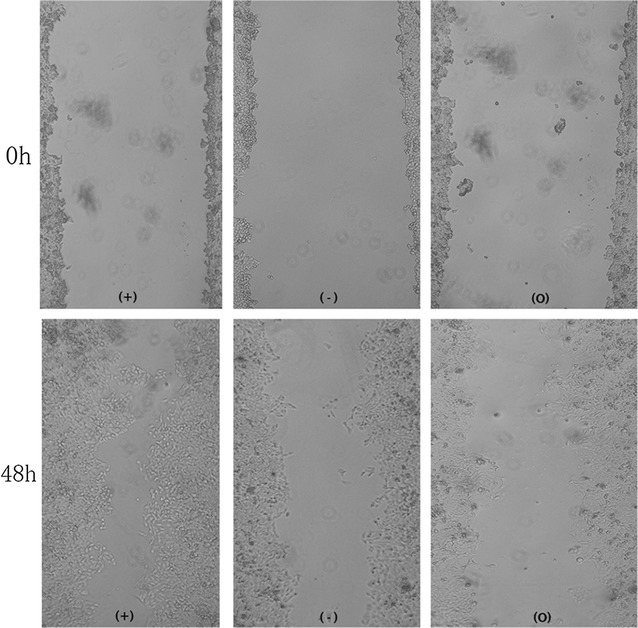
The scratch wound assay was assessed to cell motility. The exosomal 91H enhanced HCT-116 cell motility

A scratch wound assay was firstly performed to evaluated the effect of exosomal 91H on cell motility. Cells treated with cancer exosomes resulted in enhancing motility of HCT-116 cells. Furthermore, compared with infected cells with negative control, the wound recovery was significantly lower in siRNA-infected cells (Fig. 4).

**Fig. 5:**
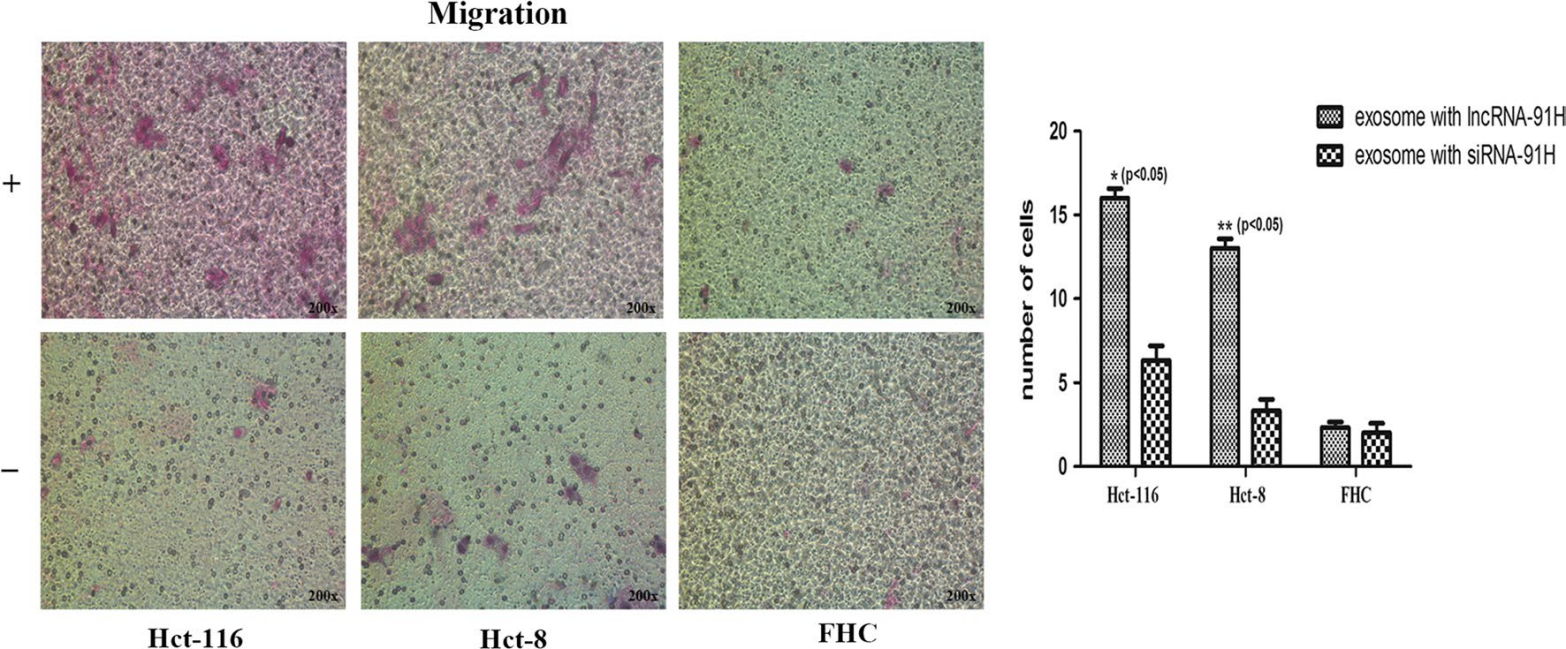
Migration assay of HCT-116, Hct-8 and FHC cells. (+): cells treated with cancer exosomes with lncRNA 91H. (−): cells treated with cancer exosomes with lncRNA 91H interference. The exosomal 91H enhanced HCT-116 and Hct-8 cells migration (P < 0.05), which had no effect on normal FHC cells migration

**Fig. 6:**
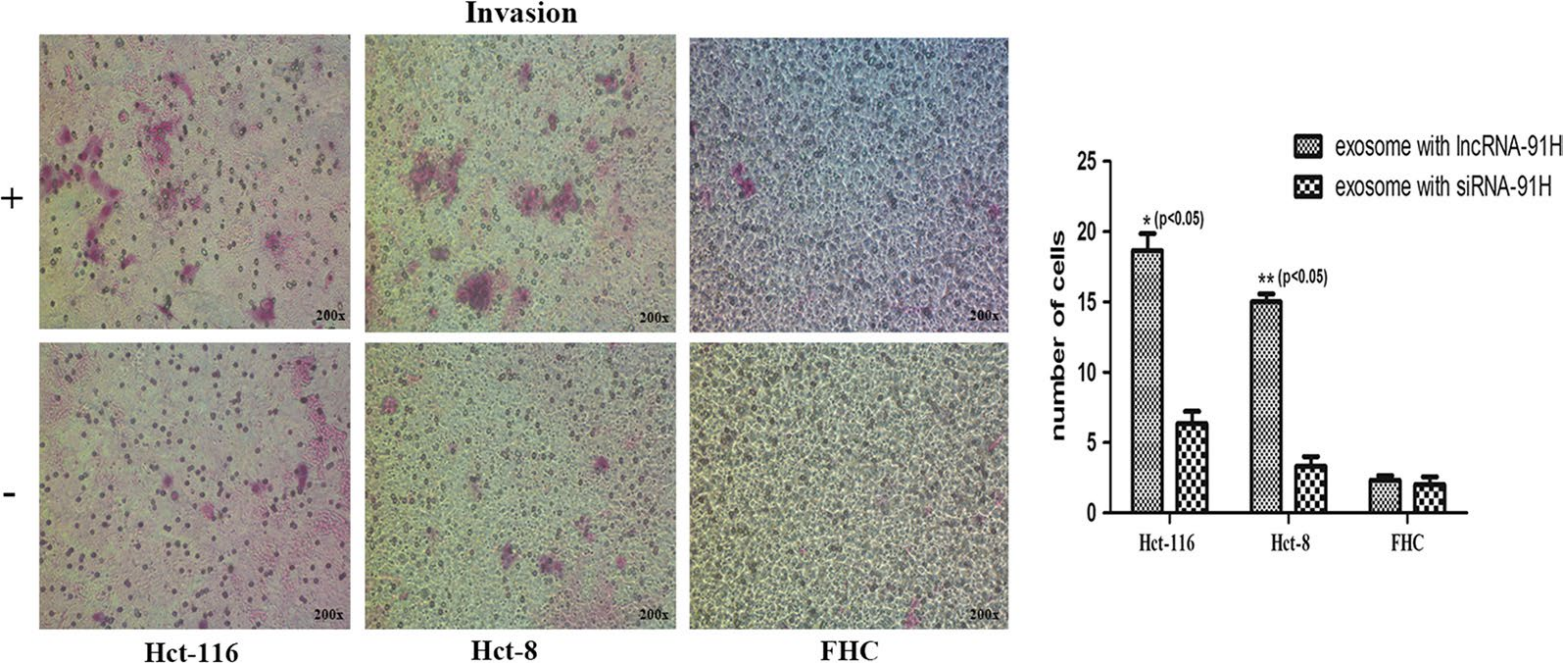
Invasion assay of HCT-116 cells Hct-8 and FHC cells. (+): cells treated with cancer exosomes with lncRNA 91H. (−): cells treated with cancer exosomes with lncRNA 91H interference. The exosomal 91H enhanced HCT-116 and Hct-8 cells invasion (P < 0.05), which had no effect on normal FHC cells invasion

In addition, migration and invasion assays were used to further gauge the effect of exosomal 91H on CRC and normal FHC cells. The results demonstrated that the migration was significantly increased in infected CRC cells with cancer exosomes, compared with negative control cells (Fig. 5, P < 0.05) and the invasiveness was also enhanced in infected CRC cells with cancer exosomes, compared with other groups (Fig. 6, P < 0.05). These findings both indicated that exosomal 91H might be closely correlated with proliferation, migration, and invasion of CRC cell lines.

### Clinical values of exosomal 91H in CRC relapse and metastasis

The exosomal 91H expression level in serum were determined by qRT-PCR for CRC patients. After half a year, the exosomal 91H expression level in 108 patients’ serum was 1.21 ± 1.24 which was higher than those who had just underwent operation (1.22 ± 0.94, P < 0.05). Furthermore, the optimal cut-off level of exosomal 91H expression for RFS was calculated by ROC curve analysis which divided the 108 CRC patients into a high 91H expression group (n = 57) with 91H expression ratio ≥ 0.85 and a low 91H expression group (n = 51) with 91H expression ratio < 0.85. Then a univariate analysis was conducted which showed that patients with higher exosomal 91H expression level usually had a higher risk in tumor relapse or metastasis than others (HR 5.13, 95% CI 1.23–21.35, P = 0.025). At last, multivariate analysis was performed in order to assess the independent prognostic factors with RFS. The results also indicated that patients with high 91H expression (HR 7.14, 95% CI 1.23–21.35, P = 0.040) were associated independently with RFS (Table 1).

**Table 1:** Cox proportional hazard analysis: impact of exosomal 91H expression and clinicopathological parameters on relapse-free survival in CRC patients

Risk factors	Category	Univariate analysis		Multivariate analysis	
		HR (95% CI)	*P*	HR (95% CI)	*P*
91H in tumor	Low (n = 51)/high (n = 57)	5.13 (1.23–21.35)	*0.025*	7.14 (1.11–46.52)	*0.040*
Sex	M (n = 84)/F (n = 24)	0.92 (0.17–4.88)	0.925	1.32 (0.12–15.23)	0.823
Age (years)	< 67 (n = 68)/≥ 67 (n = 40)	0.73 (0.17–3.16)	0.678	0.58 (0.08–4.46)	0.629
Tumor location	Colon (n = 66)/rectum (n = 42)	2.33 (0.56–9.17)	0.225	0.90 (0.46–24.28)	0.230
TNM	I–II (n = 36)/III–IV (n = 72)	4.00 (0.92–17.39)	0.065	0.93 (1.07–62.91)	*0.043*
N	N0 (n = 87)/N1–N2 (n = 21)	2.33 (0.39–14.03)	0.335	1.78 (0.20–29.35)	0.486
Grade	G1–G2 (n = 87)/G3 (n = 21)	1.89 (0.36–10.03)	0.455	2.78 (0.43–107.49)	0.174
Chemotherapy	Yes (n = 90)/no (n = 18)	1.31 (0.23–7.57)	0.765	1.00 (0.12–8.64)	0.997

Italic values indicate significance of P value (P < 0.05)

## Discussion

Despite substantial progress of lncRNAs in cancer pathogenesis, biological roles of lncRNAs are still unclear in CRC. Recently, exosomes mediated transfer of lncRNAs have been gradually demonstrated as an important regulatory mechanism in cancer development which might provide new insights into the biology of CRC [18]. To explore the biology of exosomal lncRNA in CRC, we performed this study to firstly investigate the biological roles of exosomal 91H in CRC progression and assess the effect of exosomal 91H in clinic prognosis.

In this study, remarkable high elevated levels of exosomal 91H was observed in the serum of CRC patients and cancer lines suggesting exosomal 91H may serve as a potential CRC biomarker. Then RNA interference was used to confirm the association between lncRNA 91H and cancer exosomes. Which showed that exosomal 91H levels immediately decreased when lncRNA was silenced in CRC cells. Furthermore, exosomal 91H expression of CRC patients underwent operation was also detected which showed that the exosomal 91H levels usually decreased after operation. These both indicated that tumor cells and tissues were the main sources of exosomal 91H.

Exosomes consist of a lipid bilayer, transmembrane and nonmembrane bound proteins and nucleic acids which can transfer their constituents and cargo including mRNA, miRNA, long non-coding RNAs and so on to neighbouring or distant cells with preservation of their function [19, 20]. Based on their cellular content and ability to transport content to target cells, they may provide a mechanistic link to tumor metastasis. Ramteke et al. showed that exosomes secreted by hypoxic prostate tumor cells can influence distant and neighboring tumor cells and the surrounding microenvironment which in turn enhanced prostate tumor cell invasiveness and metastasis [21]. Wei Mu had also proved that cancer exosomes also affected the host cell which strongly supported host cell migration and invasiveness [22]. Consistent with early studies’ results, we also found that CRC cells migration and invasiveness were significantly enhanced by treating with cancer exosomes with lncRNA 91H.

For migration and invasion is a prerequisite for tumor metastasis [23], the clinical values of exosomal 91H in tumor prognosis was detected in the study. The univariate and multivariate analysis results of RFS both revealed that patients with high exosomal 91H expression usually had an higher risk in tumor replace than those with low exosomal 91H expression. It was an independent prognostic factor as classical prognostic factors like TNM which might serve as an accurate biomarker for the prognosis of CRC patients.

HNRNPK is an RNA-binding protein which has been reported to be overexpressed in lots of cancers and involved in many tumor processes such as metastasis [24–26]. However, the regulatory mechanism of HNRNPK in CRC development still remains unclear. In this study, we found that HNRNPK directly interacted with lncRNA 91H in CRC cells. Its expression level was closely related to lncRNA 91H expression. HNRNPK was regulated by lncRNA 91H mediation so that it could further promote tumor development and metastasis.

## Conclusion

In summary, our results suggested that lncRNA 91H was mainly transferred by exosomes which greatly enhanced tumor migration and invasion through modifying HNRNPK expression. Moreover, exosomal 91H in cancer patients’ serum was significantly higher than healthy people and correlated with aggressive tumor replace and metastasis. These findings might provide a foundation for development of an early diagnostic and prognostic biomarker for CRC and determination of innovative therapeutic strategies.

## Abbreviations

CRC: colorectal cancer
lncRNAs: long noncoding RNAs
GC: gastric cancer
FHC: normal human intestinal epithelial cell line
ROC: receiver operating characteristic curve analysis
RFS: relapse-free survival
TEM: transmission electron microscope
HNRNPK: heterogeneous nuclear ribonucleoprotein K
